# A Greek registry of current type 2 diabetes management, aiming to determine core clinical approaches, patterns and strategies

**DOI:** 10.1186/s12902-019-0364-5

**Published:** 2019-04-25

**Authors:** Stavros Liatis, Styliani Iraklianou, Kyriakos Kazakos, George Mastorakos, Kostas Milios, Zadalla Mouslech, Marina Noutsou, Emmanouil Pagkalos, Christos Sampanis, AGREEMENT Investigators, Alexiou Zoi, Almanidou-Kougioumtzidi Olga, Anastasiou Eleni, Avramidis Iakovos, Βakidis Sofoklis, Bargiota Alexandra, Bikas Christos, Bousboulas Stavros, Boutel Dimitrios, Chaliotis Georgios, Chrisoulidou Alexandra, Didangelos Triantafyllos, Dimitriadis George, Dimou Eftihia, Douitsis Petros, Doupis John, Exiara Triada, Gkioka Maria, Gkioulos Nikolaos, Grigoropoulou Pinelopi, Ioannidis Ioannis, Kirlaki Evridiki, Kitsios Kostas, Kokkoris Panagiotis, Kotsa Kalliopi, Kouroglou Maria, Lanaras Leonidas, Magiannis Konstantinos, Manes Christos, Marathonitis Georgios, Melidonis Andreas, Migdalis Ilias, Mitrakou Asimina, Papantoniou Stefanos, Papazoglou Dimitrios, Piaditis Georgios, Potolidis Evangelos, Prokovas Ioannis, Rizos Evangelos, Rogkoti Maria, Romanidou Alexandra, Sampanis Christos, Satsoglou Aimilios, Simelidis Dimitrios, Taisir El Hasban, Tentolouris Nikolaos, Thanopoulou Anastasia, Tolis Apostolos, Tsanikidis Iraklis, Tsapas Apostolos, Tsapogas Panagiotis, Tsatsoulis Agathoklis, Tsotoulidis Stefanos, Tzatzagou Glykeria, Vasiliadis Panagiotis, Vasilopoulos Charalampos, Vlachogiannis Anestis, Vryonidou Andromachi, Xilomenos Apostolos

**Affiliations:** 1 First Department of Propaedeutic Medicine, Diabetes Center, Athens University Medical School, Laiko Hospital, Ag Thoma 17, 11527 Athens, Greece; 2 Diabetes Center, Tzanio General Hospital, Piraeus, Greece; 3 Faculty of nursing, Alexander Technological Educational Institute of Thessaloniki, Thessaloniki, Greece; 4 Department of Endocrinology, Metabolism and Diabetes, Aretaieio Hospital, School of Medicine, University of Athens, Athens, Greece; 5 Medical Department, Sanofi, Athens, Greece; 6 1st Medical Propaedeutic Department of Internal Medicine, AHEPA University Hospital, Aristotle University of Thessaloniki, Thessaloniki, Greece; 7 Diabetes Center, General Hospital of Athens “Hippokration”, Athens, Greece; 8 Clinic “Thermi,” Thessaloniki, Thessaloniki, Greece; 9 Diabetes Centre, Second Propaedeutic Department of Internal Medicine, General Hospital of Thessaloniki – “Hippokration”, Thessaloniki, Greece

**Keywords:** Type-2 diabetes mellitus, Glycaemic control, Clinical decisions, Compliance, Guidelines

## Abstract

**Background:**

To analyze data in terms of the glycaemic control and therapeutic regimens used for Type-2 Diabetes Mellitus (T2DM) management in Greece, identify factors that influence clinical decisions and determine the level of compliance of T2DM management with the latest international and local guidelines.

**Methods:**

‘AGREEMENT’ was a national-multicenter, non-interventional, cross-sectional disease registry. A total of 1191 adult T2DM patients were enrolled consecutively from 59 sites of the National Health System (NHS) or University Hospitals, representing the majority of Diabetes centers or Diabetes outpatient clinics in Greece with a broad geographic distribution. Patients were stratified by gender and analysis was done according to 3 treatment strategies: A (lifestyle changes or use of one oral antidiabetic agent), B (up to 3 antidiabetic agents including injectables but not insulin) and C (any regimens with insulin).

**Results:**

Mean (±SD) HbA1c % of the total population was 7.1 (±1.2) while mean (±SD) FPG (mg/dl) was measured at 136 (±42). The proportion of patients who achieved HbA1c < 7% was 53% and ranged from 74.2% for group A, to 60.6% for group B and 35.5% for group C. Median age of the studied population was 65.0 year old (Interquartile Range-IQR 14.0) with an equal distribution of genders between groups. Patients on insulin therapy (treatment strategy C) were older (median age: 67 years vs 63 or 65 for A and B, respectively) with longer diabetes duration (mean duration: 15.3 years vs 5.2 and 10.1 for A and B, respectively). Patients who received insulin presented poor compliance. There was a consensus for a series of decision criteria and factors that potentially influence clinical decisions, used by physicians for selection of the therapeutic strategy among the three groups. Compliance with international and Greek guidelines received a high score among groups A, B and C. No significant differences were presented as per sites’ geographic areas, NHS or University centers and physicians’ specialty (endocrinologists, diabetologists and internists).

**Conclusions:**

The presented findings suggest the need for improvement of the glycaemic control rate, especially among insulin treated patients as this group seems to achieve low glycaemic control, by setting appropriate HbA1c targets along with timely and individualised intensification of treatment as well as post-therapy evaluation of the compliance with the proposed treatment.

**Electronic supplementary material:**

The online version of this article (10.1186/s12902-019-0364-5) contains supplementary material, which is available to authorized users.

## Background

Diabetes is a multifaceted disease which causes major morbidity and mortality due to micro- and macro- vascular complications [1]. Evidence from key studies established the importance of tight and sustained glycaemic control among type 1 and 2 diabetic patients [2, 3]. Metformin monotherapy should be started at diagnosis of Type-2 Diabetes Mellitus (T2DM) along with lifestyle modifications.

Current treatment guidelines therefore advocate a patient-centred approach, with treatment goals of HbA1c < 7.0% according to the American Diabetes Association (ADA), the European Diabetes Association (EASD) [4] and the Hellenic Diabetes Association (HDA) [5] and < 6.5% according to the International Diabetes Federation (IDF) [6] and the American Association of Clinical Endocrinologists (AACE) [7] while they stress the need to modify therapy if HbA1c goal is not met within 3 months.

Nevertheless, despite treatment guidelines, a large proportion of T2DM patients achieve suboptimal goals. The frequency of inadequate glycaemic control is commonly around 50% and even as high as 76% [8–13]. In the European Study on Cardiovascular Risk Prevention and Management in Usual Daily Practice (EURIKA), HbA1C < 6.5% was reached by 37% of treated patients. Particularly, 44% of patients from Greece achieved HbA1C < 6.5% [14]. Another recent retrospective observational study in Greece showed that the proportion of patients achieving the target of HbA1c < 7% was 53.9% in 2012 versus 56.1% in 2006 [15].

In PANORAMA, an observational study of T2DM patients, assessing glycaemic control and treatment patterns, 37.4% of patients enrolled, had an HbA1c ≥ 7% with a mean HbA1c of 6.9%. Particularly 32.9% of patients from Greece did not achieve HbA1c < 7%, with a mean HbA1c of 6.7% [16].

Limited data are currently available on T2DM management, daily clinical practice and real-life treatment in Greece to the best of our knowledge. Clearly, an update on glycaemic control in the general population of T2DM patients and a possible assessment of reasons why treatment goals are not achieved is needed. The Disease Registry ‘AGREEMENT’ was designed to provide a reliable picture of current T2DM management in the public healthcare sector in Greece, aiming to determine clinical approaches, therapeutic strategies, level of compliance with latest international and local guidelines in terms of glycaemic control and therapeutic regimens used [4, 5].

## Methods

### Study design

AGREEMENT was a national-multicentre, non-interventional, cross-sectional disease registry, conducted under real life conditions of daily clinical practice of T2DM in the public healthcare sector in Greece.

In close collaboration with the Hellenic Diabetes Association (HDA), the Northern Greece Diabetes Association (NGDA) and the Hellenic Endocrine Society (HES), a Steering Committee supervised the whole procedure, advised on scientific issues, determined the recruiting capacity of each site and contributed to the analysis of results and writing of the manuscript. The Steering Committee, was composed of the authors, 8 of whom (SA, KK, SL, GM, ZM, MN, EP, CS) were indicated by the three scientific medical societies involved and 1 Sanofi staff member (KM). The data were gathered by site investigators and the sponsor performed site monitoring and data collection. The data were analysed by ANTAEA Consulting.

The study protocol was approved by all Hospitals’ Review Boards and conducted in accordance with Good Pharmacoepidemiology Practices (GCPs) and all applicable regulations. All patients provided written informed consent.

### Data sources

All patients were recruited in one single visit and all relevant data from medical records were registered in an electronic CRF (e-CRF). The study collected data for two periods, the initial diagnosis period, concerning the medical and diabetes history as well as the status of the patients and the current period (inclusion visit). Patients’ data registered in e-CRF included demographics and living conditions, anthropometric measurements including physical activity, T2DM treatment at initial diagnosis and at current period, lab measurements regarding glycemia and lipid profile, blood pressure measurements, family history of T1DM and T2DM, diet assessment, smoking and alcohol consumption, co-morbidities and overall quality of life estimations through a non validated questionnaire based on simple questions and using a scale from 1 to 10 (worst to best).

Since 70% of participants had been initially diagnosed at another centre than that participating in this study, this piece of information was used only for benchmarking and not for statistical inference. The following data were collected: T2DM treatments used currently and in the past (depending on availability of data), factors that potentially influence clinical decisions, level of compliance with latest international and local clinical guidelines in terms of glycaemic control and therapeutic regimens used, glycaemic control expressed as HbA1c < 7% and 130 mg/dl > FPG > 70 mg/dl at current visit.

### Site and patient selection

The sites selection process was performed following the response of 96 diabetic centers functioning in a public setting to a relative feasibility questionnaire. Of those, 69 sites accepted initially to participate and 59 were active at the end of the study.

Patients were included depending on the recruitment capacity of each site, in order to avoid any centers driving the results. The Steering Committee made a classification of the 69 diabetic centers, in 3 categories: high capacity (A), medium capacity (B) and low capacity (C) according to the number of patient monthly visits in each site, as recorded in the feasibility questionnaire. 18 out of the 69 sites were classified as high capacity (group A), 19 as medium capacity (group B) and 32 as low capacity (group C).

Patients were further stratified by treatment strategies using the information of a market survey as the main source of real life stratification, [regarding the therapeutic strategies for the diabetic patients]. 20% of patients with lifestyle changes or receiving up to one oral anti-diabetic agent (treatment strategy A), 40% of patients receiving 2 or 3 antidiabetic agents including injectables but not insulin (treatment strategy B) and 40% of patients receiving insulin with or without other anti-diabetic medication (treatment strategy C). Patients were finally stratified according to gender by 50%.

Stratification of patients took place according to the breakdown of Additional file 1: Table S1. Patients were recruited in a sequential manner, minimizing patient selection bias. Data on antidiabetics medication including mean dose in total and per treatment strategy are provided in Additional file 1: Table S2a-e.

### Study population

A total of 1236 T2DM patients from 69 sites were planned to be enrolled in the study. T2DM patients ≥18 years old, were eligible. Exclusion criteria included T1DM patients, pregnant or lactating women, any clinically significant acute major organ or systemic disease and need for hospitalization occurring within 3 months before enrolment. All participants provided written informed consent.

### Study objectives

The primary objectives were the collection and analysis of data on current clinical practice and relevant treatment strategies, the identification of factors that potentially influence clinical decisions, the level of compliance with latest international and local guidelines in terms of glycaemic control and use of therapeutic regimens based on answers to relevant questionnaires of the e-CRF. Secondary objectives included potential differentiation in T2DM management between geographic areas, National Health System- NHS and University centres and different medical specialties (endocrinologists, diabetologists and internists).

### Sample population size

The study planned to include approximately 1200 patients. This would allow to calculate the percentage of patients with HbA1c < 7%, both in total and in different subgroups, with an acceptable precision, based on the fact that the expected prevalence of different therapeutic strategies would be close to relevant data for categorization of treatment approaches and the expected percentage of patients achieving HbA1c < 7%. The expected patients with HbA1c < 7% is close to 50% [11, 13–15].

### Subgroups of interest

Study population was analysed according to three treatment strategies. In addition patients were grouped according to HbA1c < 7% or ≥ 7% or < 6.5, 6.5–7.0%, < 7.0, 7.0–7.5%, 7.5–8.0% and > 8.0% and FPG of 70-100 mg/dl, 100-130 mg/dl and ≥ 130 mg/dl.

### Statistical analysis

Descriptive statistics, Chi-square test for testing differences between strategies and Kruskal – Wallis test to test hypotheses when distributions were not normal, were the main statistical methods used. In the descriptive analysis, the variables were presented as mean, median and the quartiles. Dispersants measures were calculated by variance and standard deviation and IQR. No sensitivity analysis was performed. Statistical analysis SAS Enterprise Guide was used. There was no record of current treatment strategy for 2 patients who were excluded from the analysis.

## Results

### Patient characteristics

**Fig. 1:**
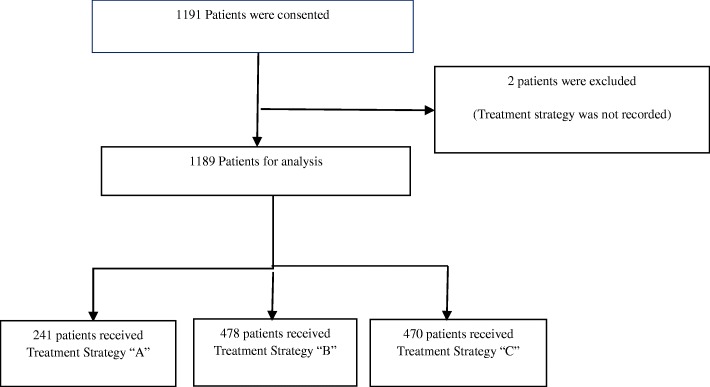
Study Flow chart

Overall 1189 patients (out of 1191 initially consented) from 59 sites, were recruited between 13 June 2014 (first subject in) and 04 June 2015 (last subject in). Patients’ flowchart at current visit is shown in Fig. 1. Median age of the studied population was 65.0 year old (IQR 14.0) with an equal gender distribution between the groups. Demographics analyses by treatment strategies A, B, and C revealed statistical significant differences as indicated in Table 1. The difference in patients’ age and in years under treatment between groups were statistically significant (K-W test, *p* = 0.002, K-W test, *p* <  0.001). Patients that received insulin (treatment strategy C) were older (median age 67 years vs 63 or 65 for A and B, respectively) and their median length of time under treatment was longer (15 years vs. 4 or 9 for A and B, respectively).

**Table 1:** Patients demographics, lab measurements at current period and living conditions per treatment strategy

Variable	Total	Treatment Strategy			*P*
		A	B	C	
Patients n (%)	1189 (100%)	241 (20.3%)	478 (40.2%)	470 (39.5%)	
Sex n (%)					
Male	601 (50.5%)	125 (51.9%)	241 (50.4%)	235 (50%)	0.856 ^a^
Female	588 (49.5)	116 (48.1%)	237 (49.6%)	235 (50%)	
Diabetes Duration (years) (mean ± SD)	11.2 ± 8.6	5.2 ± 5.7	10.1 ± 7.5	15.3 ± 8.6	
Years under Treatment (median – IQR)	10 (4–16)	4 (1–7)	9 (4–14)	15 (9–20)	< 0.001 ^b^
Age (years) (median - IQR)	65 (58–72)	63 (56–70)	65 (58–72)	67 (58–73)	0.002 ^b^
Age group					
< 60	362 (30.4%)	89 (36.9%)	139 (29.1%)	134 (28.5%)	0.041 ^a^
60–70	477 (40.2%)	97 (40.2%)	198 (41.4%)	182 (38.7%)	
> 70	350 (29.4)	55 (22.8%)	141 (29.5%)	154 (32.8%)	
Height (cm) (median – IQR)	165 (158–172)	165 (158–172)	165 (158–172)	165 (158–172)	
Weight (kg) (median – IQR)	82 (73–93)	80 (70–90)	82 (73–92.5)	83 (73–96)	
Body Mass Index (kg/m^2^) (median – IQR)	30 (26.75–33.8)	29.4 (26.6–32.6)	30 (26.8–33.7)	30.1 (26.6–34.7)	
Systolic blood pressure (mmHg) (median – IQR)	130 (120–140)	130 (120–140)	130 (120–140)	134 (125–145)	0.03 ^b^
Diastolic blood pressure (mmHg) (median – IQR)	80 (70–85)	80 (70–85)	80 (70–84)	80 (70–83)	
eGFR (mL/min/1.73 m2) (median – IQR)	73.9 (60.6–93.2)	78.1 (59.9–96.1)	74.3 (62.0–94.5)	73.1 (60.4–89.3)	< 0.001^b^
Microalbumin urine (mcg/mg creatinine) (median – IQR)	18 (10–45)	14 (7–30)	16 (9–40)	24 (11–78.4)	< 0.001^b^
Total Serum Cholesterol (mg/dl) (median – IQR)	173 (150–200)	183 (156–204)	174 (152–201)	166 (146–193)	< 0.001^b^
Serum Triglycerides (mg/dl) (median – IQR)	127 (95–174)	126 (96–176)	125.5 (95–175)	127.5 (94.5–171.5)	
Serum LDL-C (mg/dl) (median – IQR)	97 (76–120)	104 (81–125)	96 (75–121)	92 (74–114)	0.03^b^
Serum HDL-C (mg/dl) (median – IQR)	46 (39–56)	47 (41–57)	48 (39–57)	45 (39–53)	
Marital status n (%)					
Married	1003 (84.4%)	202 (83.8%)	410 (85.8%)	391 (83.2%)	0.679^a^
Unmarried	56 (4.7%)	15 (6.2%)	18 (3.8%)	23 (4.9%)	
Divorced	39 (3.3%)	9 (3.7%)	13 (2.7%)	17 (3.6%)	
Widower / Widow	91 (7.7%)	15 (6.2%)	37 (7.7%)	39 (8.3%)	
Living Status n (%)					
Lives with his / her family	1066 (89.7%)	214 (88.8%)	431 (90.2%)	421 (89.6%)	0.848^a^
Lives alone	123 (10.3%)	27 (11.2%)	47 (9.8%)	49 (10.4%)	
Resident n (%)					
Village	237 (19.9%)	48 (19.9%)	93 (19.5%)	96 (20.4%)	0.689^a^
Provincial town	133 (11.2%)	32 (13.3%)	44 (9.2%)	57 (12.1%)	
Provincial city	179 (15.1%)	37 (15.4%)	79 (16.5%)	63 (13.4%)	
Prefectural capital	314 (26.4%)	61 (25.3%)	124 (25.9%)	129 (27.4%)	
Urban center	326 (27.4%)	63 (26.1%)	138 (28.9%)	125 (26.6%)	
Self-care Capability n (%)					
Capable of self-care	1139 (95.8%)	238 (98.8%)	462 (96.7%)	439 (93.4%)	**0.009** ^a^
Needs help sometimes	45 (3.8%)	3 (1.2%)	15 (3.1%)	27 (5.7%)	
Dependent	5 (0.4%)	0 (0.0%)	1 (0.2%)	4 (0.9%)	
Access to Health Services n (%)					
Difficult	15 (1.3%)	4 (1.7%)	4 (0.8%)	7 (1.5%)	0.914^a^
Occasional	68 (5.7%)	15 (6.2%)	29 (6.1%)	24 (5.1%)	
Easy	402 (33.8%)	83 (34.4%)	166 (34.7%)	153 (32.6%)	
Regular	440 (37%)	88 (36.5%)	179 (37.4%)	173 (36.8%)	
Systematic	264 (22.2%)	51 (21.2%)	100 (20.9%)	113 (24.0%)	
Education n (%)					
Illiterate	59 (5%)	10 (4.1%)	25 (5.2%)	24 (5.1%)	**0.032** ^a^
Basic education	592 (49.8%)	115 (47.7%)	224 (46.9%)	253 (53.8%)	
Secondary education	365 (30.7%)	67 (27.8%)	165 (34.5%)	133 (28.3%)	
Higher education	173 (14.6%)	49 (20.3%)	64 (13.4%)	60 (12.8%)	
Occupation n (%)					
Manual work	239 (20.1%)	50 (20.7%)	94 (19.7%)	95 (20.2%)	0.101^a^
Office work	151 (12.7%)	39 (16.2%)	68 (14.2%)	44 (9.4%)	
Intellectual work	26 (2.2%)	7 (2.9%)	11 (2.3%)	8 (1.7%)	
Pensioner	621 (52.2%)	109 (45.2%)	248 (51.9%)	264 (56.2%)	
Unemployed	152 (12.8%)	36 (14.9%)	57 (11.9%)	59 (12.6%)	
Financial Status n (%)					
NA	5 (0.4%)	2 (0.8%)	0 (0.0%)	3 (0.6%)	0.324^a^
Indigent	14 (1.2%)	4 (1.7%)	5 (1.0%)	5 (1.1%)	
Poor	171 (14.4%)	37 (15.4%)	55 (11.5%)	79 (16.8%)	
Moderate financial status	891 (74.9%)	173 (71.8%)	376 (78.7%)	342 (72.8%)	
Financial comfort	92 (7.7%)	21 (8.7%)	37 (7.7%)	34 (7.2%)	
Wealthy	16 (1.3%)	4 (1.7%)	5 (1.0%)	7 (1.5%)	
Insured n (%)					
No	24 (2%)	6 (2.5%)	10 (2.1%)	8 (1.7%)	0.771^a^
Yes	1165 (98%)	235 (97.5%)	468 (97.9%)	462 (98.3%)	

^a^Chi – square independence test,

^b^Kruskal Wallis test

Kruskal Wallis post-hoc pairwise comparisons between groups

• Age vs Treatment Group:

Treatment Group A vs Treatment Group C: *p* = 0.003 < 0.05

•Years under treatment vs Treatment Group:

Treatment Group A vs Treatment Group B: *p* < 0.001.

Treatment Group A vs Treatment Group C: *p* < 0.001.

Treatment Group C vs Treatment Group B: *p* < 0.001

•Systolic blood pressure vs Treatment Group:

Treatment Group A vs Treatment Group C: *p* = 0.001 < 0.05.

Treatment Group C vs Treatment Group B: *p* = 0.044 < 0.05.

•eGFR vs Treatment Group:

Treatment Group A vs Treatment Group C: *p* < 0.001.

Treatment Group C vs Treatment Group B: *p* = 0.002 < 0.05.

• Microalbumin urine vs Treatment Group:

Treatment Group A vs Treatment Group C: *p* < 0.001.

Treatment Group C vs Treatment Group B: *p* = 0.003 < 0.05

•Total Serum Cholesterol vs Treatment Group:

Treatment Group A vs Treatment Group C: *p* < 0.001.

•Serum LDL – C vs Treatment Group

Treatment Group A vs Treatment Group C: *p* = 0.002 < 0.05

Values captured in bold are significant

Basal insulin regimens were prescribed for 80% of patients, with 51% on basal insulin and OADs and 28% on basal-plus regimens. Premixes were prescribed for 19% of patients with any regimens with insulin.

There were no differences in vital signs or lab measurements such as cholesterol, triglycerides etc. among treatment groups. Living condintion characteristics were the same between treatment strategies, except education (chi square test, *p* = 0.032) and self –care capability (chi square test, *p* = 0.009). More patients of treatment strategy C (53.8%) had received only basic education as compared to those of groups A (47.7%) and B (46.9%) (p = 0.032) and needed help sometimes in terms of self-care capability (Table 1).

Considering risk factors by treatment strategy at current period (Additional file 1: Table S3), statistical significant differences were found for limited physical exercise (chi square test, *p* = 0.012) and overall poor diet (chi square test, *p* = 0.002). Patients under treatment strategy C had limited physical exercise and followed an overall poorer diet when compared to groups A and B.

Table 2 presents the reported diabetes complications by treatment strategy. Patients following treatment strategy C had more chronic complications than A and B. Acute complications and hospitalizations were rare in all therapeutic strategies.

**Table 2:** Diabetes Complications per treatment strategy at current period

Complication n (%)	Total	Treatment Strategy			*P*
		A	B	C	
Acute complications	15 (3.7%)	2 (0.5%)	1 (0.2%)	12 (2.9%)	NA
Non-ketotic Hyperglycaemic Hyperosmolar Coma	2 (0.2%)	1 (0.4%)	0 (0%)	1 (0.2%)	NA
Infections	2 (0.2%)	0 (0%)	0 (0%)	2 (0.4%)	NA
Diabetes-associated Hospitalisation	3 (0.3%)	0 (0%)	0 (0%)	3 (0,6%)	NA
Other	7 (0.6%)	1 (0.4%)	1 (0.2%)	5 (1.1%)	NA
Chronic complications	386 (32.5%)	29 (12.0%)	130 (27.2%)	227 (48.3%)	
Diabetic Retinopathy	141 (11.9%)	6 (2.5%)	36 (7.5%)	99 (21.1%)	< 0.001
Diabetic Nephropathy	140 (11.8%)	16 (6.6%)	43 (9.0%)	81 (17.2%)	< 0.001
Diabetic Neuropathy	136 (11.4%)	7 (2.9%)	33 (6.9%)	96 (20.4%)	< 0.001
Diabetic Foot: ulcers	14 (1.2%)	1 (0.4%)	6 (1.3%)	7 (1.5%)	NA
Diabetic Foot: extensive superficial lesions	7 (0.6%)	1 (0.4%)	3 (0.6%)	3 (0.6%)	NA
Diabetic Foot: gangrene	2 (0.2%)	0 (0%)	0 (0%)	2 (0.4%)	NA
Diabetic Foot: lower extremity amputation	3 (0.3%)	0 (0%)	0 (0%)	3 (0.6%)	NA
Cardiovascular events	69 (5.8%)	4 (1.7%)	29 (6.1%)	36 (7.7%)	0.005
Carotid Artery Disease	24 (2.0%)	3 (1.2%)	2 (0.4%)	19 (4.0%)	< 0.001
Peripheral Arterial Disease	48 (4%)	3 (1.2%)	12 (2.5%)	33 (7%)	< 0.001
Other	23 (1.9%)	2 (0.8%)	7 (1.5%)	14 (3%)	0.09

70% of patients declared that the initial diagnosis was made at another centre and 67% of patients by a public health sector physician.

### Collection and analysis of data on current clinical practice in T2DM management and relevant treatment patterns in public sector in Greece

Laboratory measurements of glycaemic control by treatment strategy at the time of diagnosis (initial period) and at the inclusion visit (current period) are shown in Table 3. HbA1c and FPG values were available for both periods while PPG values were recorded only at the inclusion visit.

**Table 3:** Initial diagnosis and current period lab measurements of glycaemic control per treatment strategy

Variable	Initial diagnosis				Current period				
	Treatment Strategy				Treatment Strategy				*P*
	All	A	B	C	All	A	B	C	
Patients n (%)	1189 (100%)	722 (60.7%)	334 (28.1%)	133 (11.2%)	1189 (100%)	241 (20.3%)	478 (40.2%)	470 (39.5%)	
Full analysis set, n	572 (100%)	359 (62.8%)	151 (26.4%)	62 (10.8%)	1120 (100%)	225 (20.1%)	452 (40.4%)	443 (39.6%)	
HbA1c %									
Median (IQR)	7.8 (7.0–9.0)	7.5 (6.8–8.2)	8.6 (7.7–10.0)	10.0 (8.5–11.7)	6.9 (6.3–7.5)	6.4 (6.0–7.0)	6.7 (6.3–7.2)	7.3 (6.7–8.0)	< 0.001^b^
Mean ± SD	8.4 ± 1.4	7.5 ± 1.6	8.3 ± 1.1	9.3 ± 2.2	7.1 ± 1.2	6.6 ± 1.0	6.9 ± 1.1	7.5 ± 1.2	
Adjusted^c^ mean ± SD		8.1 ± 0.2	8.3 ± 0.1	8.5 ± 0.1		7.0 ± 0.1	7.0 ± 0.1	7.2 ± 0.1	0.771
< 6.5%	42 (7.0%)	38 (10.0%)	3 (1.0%)	1 (1.0%)	339 (30.0%)	115 (51.1%)	159 (35.2%)	65 (14.7%)	< 0.001^a^
[6.5–7.0%)	73 (12.0%)	65 (18.0%)	8 (5.0%)	0 (0%)	259 (23.0%)	52 (23.1%)	115 (25.4%)	92 (20.7%)	
< 7.0%	115 (19.0%)	103 (28.0%)	11 (6.0%)	1 (1.0%)	598 (53.0%)	167 (74.2%)	274 (60.6%)	157 (35.4%)	
[7.0–7.5%)	92 (16.0%)	73 (20.0%)	16 (10.0%)	3 (4.0%)	218 (19.0%)	34 (15.1%)	94 (20.8%)	90 (20.3%)	
[7.5–8.0%]	119 (20.0%)	81 (22.0%)	30 (19.0%)	8 (12.0%)	149 (13.0%)	14 (6.2%)	42 (9.3%)	93 (21.0%)	
> 8.0%	246 (43.0%)	102 (28.0%)	94 (62.0%)	50 (80.0%)	155 (13.0%)	10 (4.5%)	42 (9.3%)	103 (23.3%)	
FPG mg/dl									
Median (IQR)	177 (142–234)	160 (135–200)	200 (160–259)	240 (186–350)	126 (110–150)	120 (107–135)	126 (110–150)	130 (111–161)	< 0.001^b^
Mean ± SD	201 ± 85	179 ± 63	221 ± 82	280 ± 133	136 ± 42	126 ± 32	135 ± 41	141 ± 46	
Adjusted^c^ mean ± SD		189.4 ± 6.9	201.1 ± 4.7	206.3 ± 5.1		134.8 ± 3.0	133.1 ± 2.0	138.6 ± 2.1	0.180
Median (IQR)	NA	NA	NA	NA	144 (130–160)	152 (138–178)	170 (145–200)	144 (130–160)	< 0.001^b^
Adjusted^c^ mean ± SD	NA	NA	NA	NA		163.2 ± 3.7	166.0 ± 2.5	169.6 ± 2.6	0.375

^a^Chi – square independence test,

^b^Kruskal Wallis test

^c^Mean values adjusted for age, duration of diabetes, body mass index and family history of diabetes

Kruskal Wallis post-hoc pairwise comparisons between groups

•HbA1c % vs Treatment Group:

Treatment Group A vs Treatment Group B: *p* < 0.001.

Treatment Group A vs Treatment Group C: *p* < 0.001.

Treatment Group C vs Treatment Group B: *p* < 0.001

•FBG vs Treatment Group:

Treatment Group A vs Treatment Group B: *p* = 0.003

Treatment Group A vs Treatment Group C: *p* < 0.001.

**Fig. 2:**
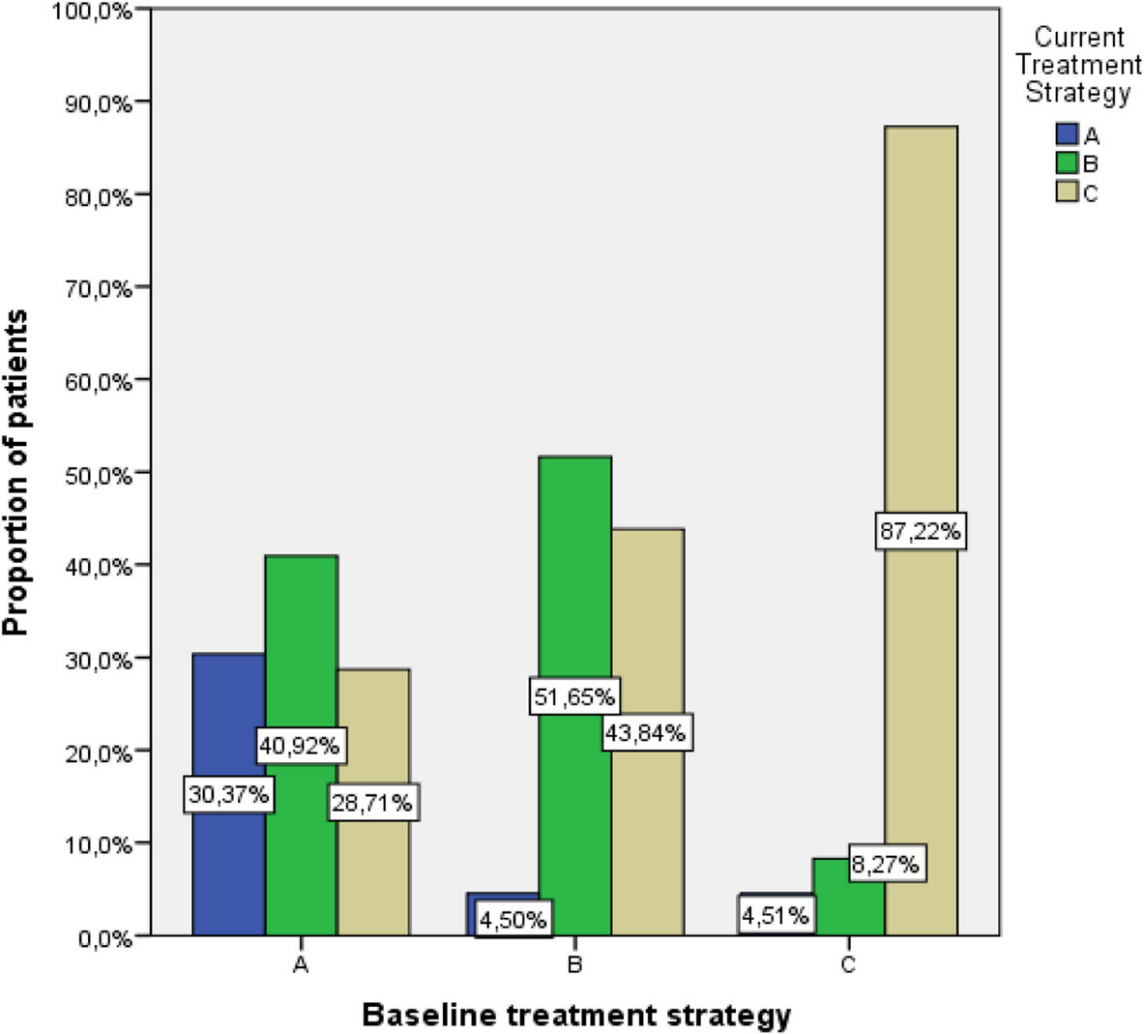
Percentage (%) of patients that changed treatment strategy between initial diagnosis and current period

Approximately 30% of patients under treatment strategy A, 50% of patients under B and 90% of patients under C at the time of diagnosis continued the same treatment regimen at current period, as shown in Fig. 2.

Mean (±SD) HbA1c % and FPG (mg/dl) of the total population decreased from 8.7 ± 7.5, 201 ± 85 at the time of diagnosis (initial period) to 7.1 ± 1.2, 136 ± 42 at the inclusion visit (current period).

At the inclusion visit (current period), mean (±SD) HbA1c % and FPG (mg/dl) values were higher among patients under treatment strategy C 7.5 ± 1.2, 141 ± 46 followed by patients under B 6.9 ± 1.1, 135 ± 41 and A 6.6 ± 1.0, 126 ± 32 (Chi square test, *p* <  0.001). PPG values were higher among patients under treatment strategy B followed by patients under A and C. However, after adjustment for covariates that are important predictors of T2DM progression, such as BMI, age, duration of DM and family history of DM, the difference in HbA1c and FPG between treatment groups were attenuated, not reaching any more statistical significance (Table 3).

**Fig. 3:**
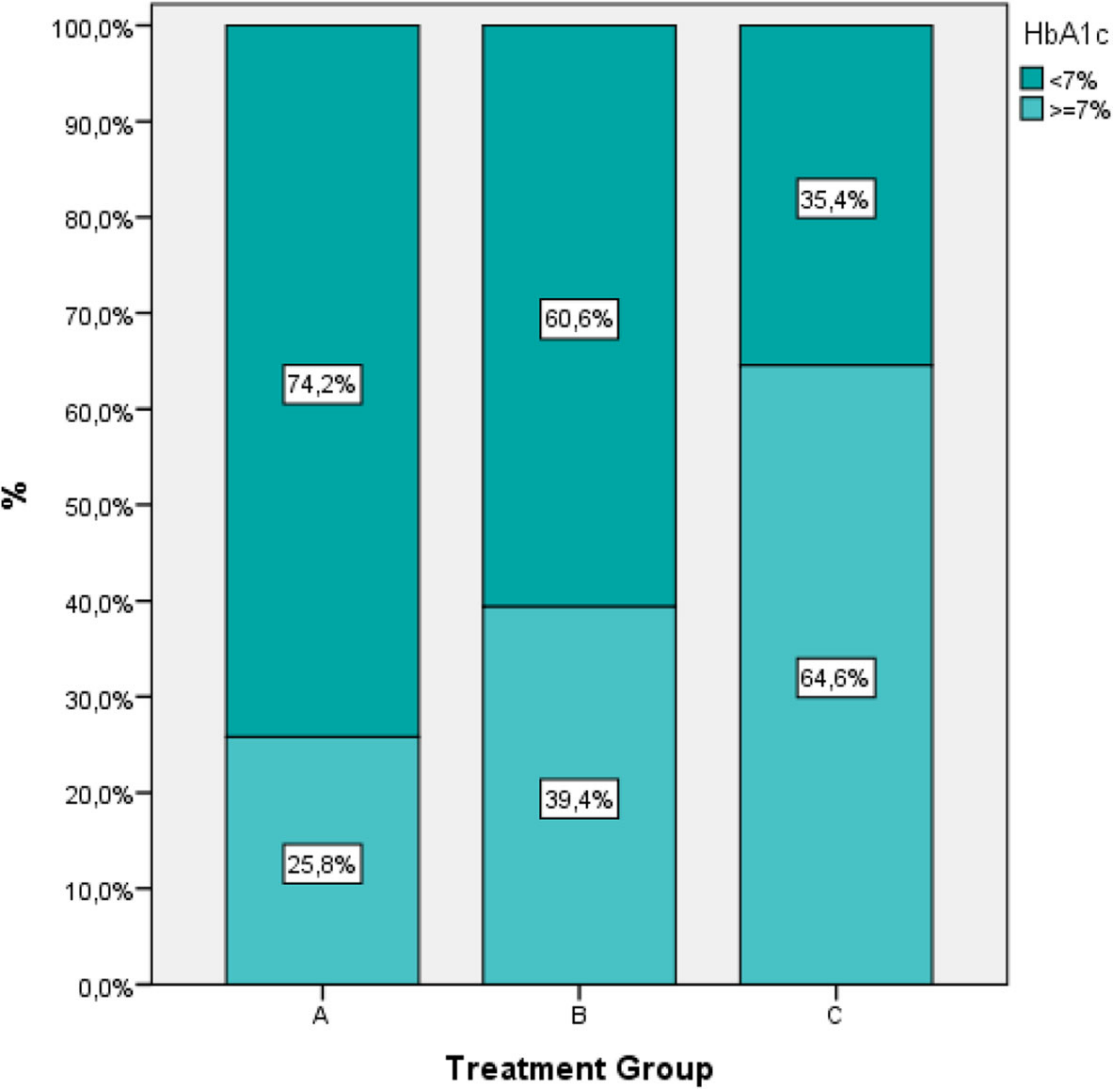
Percentage (%) of patients at HbA1c < 7% per treatment strategy at current period

**Fig. 4:**
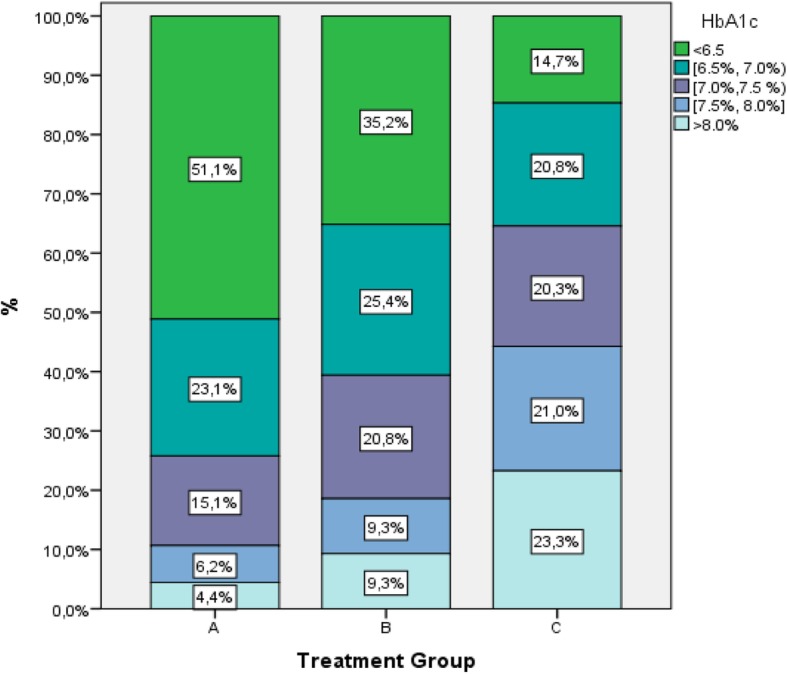
HbA1c values between < 6.5, 6.5–7.0%, 7.0–7.5%, 7.5–8.0%, > 8.0% per treatment strategy at current period

Overall, 53% (95% CI: 50.2–55.8%) of patients had HbA1c < 7% at the inclusion visit (current period). The proportion of patients with HbA1c < 7% ranged from 74.2% (95% CI: 68.7–79.7%) for A, to 60.6% (95% CI: 56.2–65.0%) for B and 35.4% (95% CI: 31.2–39.2%) for C. (Table 3, Fig.3). Distribution of HbA1c values across < 6.5, 6.5–7.0%, 7.0–7.5%, 7.5–8.0%, > 8.0% between different treatment strategies are presented in Fig. 4 (Chi square test, *p* <  0.001).

Considering the targets of HbA1C for each patient the time of diagnosis (initial period) and the goal achievement at the inclusion visit (current period), 58.1% of patients under treatment strategy A achieved the goal set compared to 52.5% of B and 40.2% of C (Pearson Chi-Square test, *p* = 0.000).

Distribution of FPG values across < 70 mg/dl, 70-100 mg/dl, 100-130 mg/dl and ≥ 130 mg/dl between different treatment strategies are also presented in Additional file 1: Table S4.

The improvement of laboratory measurements of HbA1c and FPG values at the inclusion visit (current period) compared to their respective measurements at the time of diagnosis (initial period) is presented in Additional file 1: Table S5. Treatment strategy C had the greatest improvement for both HbA1c and FPG.

The assessment of patients’ compliance, interest and active participation per therapeutic strategy is presented in Additional file 1: Table S6. 7% of all patients declared poor compliance with the prescribed medication. 9.1% of patients who followed treatment strategy C were less compliant while differences between treatment strategies were statistically significant (*p* <  0.001). 71.9% of patients overall had a satisfactory interest and an active participation in the treatment of the disease.

Using a scale from 1 to 10 (Worst to Best), patients assessed subjectively their Quality of Life (QoL), at each period (Additional file 1: Table S7). QoL score was improved for treatment strategies A and B and remained the same for C. Using Kruskal – Wallis test to examine whether there are statistically significant differences between the three treatment groups, the hypothesis was rejected (p <  0.001).

### Identification of factors that potentially influence clinical decisions and guide management strategies in T2DM

Using a scale from 1 to 10 (completely disagree to completely agree), Table 4 presents the score for decision criteria used for selection of therapeutic strategy. For the three treatment strategies there was a consensus (8–10) for all decision criteria while the differences in responses between the therapeutic strategies were statistically significant. Factors such as compliance with international and local guidelines, physician’s clinical experience and familiarity, compliance with drug indications and prescribing guidelines, efficacy and safety of each treatment strategy with minimization of adverse reactions received the highest score among groups A, B and C.

**Table 4:** Decision criteria per treatment strategy at current period

Decision criteria (Score 1–10)	Treatment Strategy						*P*
	A		B		C		
	Median	IQR	Median	IQR	Median	IQR	
Adjustment to patient’s peculiarities, principles / values & personal characteristics	10	(9–10)	9	(8–10)	8	(7–10)	< 0.01
Simplicity and convenience of treatment regimen	10	(9–10)	9	(8–10)	8	(7–9)	< 0.01
Compliance with International and Greek Guidelines for the management of DM-2	10	(9–10)	10	(8–10)	9	(8–10)	< 0.01
Choice due to corresponding physician’s clinical experience and / or familiarity	10	(9–10)	9	(8–10)	9	(8–10)	< 0.01
Compliance with the specific administration indications of the formulations	10	(9–10)	9	(8–10)	9	(8–10)	< 0.02
Compliance with Prescribing Guidelines from Regulatory Agencies or Insurance Funds	10	(8–10)	9	(8–10)	9	(8–10)	< 0.01
Greater Efficacy of the specific treatment	10	(9–10)	9	(8–10)	9	(8–10)	< 0.02
Greater Safety of the specific treatment	10	(9–10)	9	(8–10)	9	(8–10)	0.01
Better efficacy / safety combination with the specific treatment	10	(9–10)	9	(8–10)	9	(8–10)	< 0.01
Pursuing the lowest possible cost for the patient	10	(8–10)	8	(7–10)	8	(6–9)	< 0.01
Pursuing the lowest possible burden for Government Funds	10	(8–10)	8	(6–10)	8	(6–9)	< 0.01
Better financial cost and clinical benefit (efficacy) ratio	10	(8–10)	9	(8–10)	8	(7–9)	< 0.01
Minimization of adverse reactions from treatment	10	(9–10)	9	(8–10)	9	(8–10)	< 0.01

### Level of compliance of current clinical management of T2DM patients in Greece with latest international and local clinical practice guidelines in terms of glycaemic control and therapeutic regimens used

Compliance with international and Greek guidelines for the management of T2DM received a high score among groups A, B and C as presented in Table 4.

### Identification of any differentiations in clinical practice in T2DM management between different geographic areas in Greece, between NHS and university centres and between physicians of different medical specialties

No significant differences between groups were identified when analysing each treatment strategy as per sites’ geographic areas, management between NHS or University centres and physicians’ specialty (endocrinologists, diabetologists, and internists) as presented in Additional file 1: Table S8.

### Safety data collection

Spontaneous reporting of Adverse Events (AEs) and safety data collection were made. Investigators were advised to report any AE or Serious AEs (SAE) to the Marketing Authorization Holder of the suspected pharmaceutical product or to the National Organization of Medicines via the completion of the yellow card. No AEs were reported during the study.

## Discussion

The AGREEMENT study provides an important update and snapshot of real world glycaemic control in Greece. HbA1c values of 1189 T2DM patients receiving initial treatment have decreased substantially during the time interval between the time of diagnosis (initial period) and the inclusion visit (current period).

An overall mean decrease of HbA1c of 1.6% from 8.7 to 7.1% was observed. The rate of overall glycaemic control defined as HbA1c < 7% was 53% at the inclusion visit (current period) with differences between treatment groups. 74.2% of patients of treatment strategy A had HbA1c < 7%, compared to 60.6% of B and 35.4% of C.

The poorer glycaemic control with insulin treatment was possibly related to the disease progression and the fact that clinicians prescribe insulin to patients with most advanced diabetes. There is a number of barriers that may prevent initiation and optimization of insulin therapy including fear/risk of hypoglycaemia, lack of dose titration and flexibility, poor adherence and persistence, weight gain and treatment satisfaction [17–22]. These barriers must be mitigated to improve diabetes management. Patients with most advanced diabetes may need an additional treatment such as rapid acting insulin (RAI) on top of basal insulin.

An age effect was also identified with insulin treated patients since these were older and with longer time under treatment. 83.8% of elderly insulin treated patients above 65 years old reached HbA1c ≤ 8%. Moreover they had limited physical exercise, followed a poorer diet and presented poorer compliance with treatment. Older age and presence of comorbidities may influence the target for less stringent glucose control as reflected by HbA1c according to current ADA/EASD treatment guidelines [4]. QoL score was improved for treatment strategies A and B and remained the same for C. This is in contrast with Mellita study, a 6 month observational study in everyday clinical practice in Greece, that showed high compliance rate with the addition of insulin glargine on inadequately controlled T2DM patients with oral antidiabetic drugs (OADs) and benefits in both glycaemic control and health related QoL [23]. Population characteristics and changes in clinical outcomes among insulin treated patients may explain the differences in QoL score between Agreement and Mellita studies.

In this study, the percentage of patients not achieving the cut-off value of HbA1c < 7% was approximately 47% of the participants. Our results are in agreement with other similar observational studies [14–16, 24].

The reported 60.6% of patients in AGREEMENT study receiving up to 3 antidiabetic agents including injectables but not insulin and achieving HbA1C < 7%, was consistent with the results of another study, where 59% of patients with different non-insulin drugs achieved HbA1c < 7% followed by HbA1c reduction of approximately 1% within 6 months [25].

Compliance with international and Greek guidelines received a high score among groups A, B and C in terms of glycaemic control and therapeutic regimens used. No significant differences were presented when analysing as per sites’ geographic areas, management between NHS or University centres and physicians’ specialty.

Our real life observational study has both strengths and weaknesses. The strength of this observational study is that it examined every day clinical practice in a large sample of patients, indicating a degree of representativeness and extrapolation of available management of T2DM to the entire population in the public sector.

Certain weaknesses can be raised since the exact time elapsed from the time of diagnosis till current period was not predetermined from the study protocol. Also there was no information recorded on medical records concerning hypoglycaemia episodes per treatment strategy. This emphasises the need for better registration of hypoglycaemic episodes in this patient population by sites.

Limitations also concerned the non-randomised study design, which does not allow to establish any causal relationship between exposure and outcomes. Insulin titration and diet modification were left to the clinical judgement of the physicians and blood samples were measured locally. Improvement of the QoL Score and compliance was based on patients’ answer to simple questions and not to a validated questionnaire thus limiting the comparability of patient reported outcomes to the results of other studies. Another limitation of the current study is that it reflects clinical practice 3–4 years ago as patients were recruited between June 2014 and June 2015.

## Conclusion

In conclusion, the present nation-wide observational study extended our understanding of the T2DM management in the public sector in Greece.

Still a substantial proportion of T2DM patients (47%) did not achieve recommended HbA1c targets and this was particularly true in the most advanced and older patients. Clinical inertia exists in diabetes care resulting in suboptimal glycaemic control. Among different treatment strategies, the proportion of patients with HbA1c < 7% ranged from 74.2% for A, to 60.6% for B and 35.4% for C. Moreover it seems that there was a failure to achieve individual targets set for each patient by treatment strategies. 41.9% of patients under treatment strategy A failed to achieve the target set compared to 47.5% of B and 59.8% of C. Insulin treated patients were older, had more complications, limited physical exercise and lower overall adherence to diet.

There was a consensus of factors that potentially influence clinical decisions and treatment strategies, a high degree of compliance with international and local guidelines and no differentiations were noted in T2DM management between geographic areas, National Health System- NHS and University centres and different medical specialties.

Our study findings suggest there is a room for improvement of glycaemic control rate and call for further activities and educational awareness campaigns by all parties involved in diabetes management in the public domain in Greece to help patients to achieve better glycaemic control, to optimize the timing of add-on therapies and eventually minimize the risk of complications in diabetic population. Particular attention must be given among insulin treated patients to improve their clinical outcomes as this group seems to achieve low glycaemic control. This can be done by setting appropriate HbA1c targets along with timely and individualised intensification of treatment to ameliorate disease outcomes as well as post-therapy evaluation of the compliance with the proposed treatment. Further studies to explore the level of glycaemic control and its associated factors in the private sector will further contribute to evaluate disease management strategies.

## Additional file

Additional file 1: AGREEMENT Investigators. **Table S1.** Stratification of patients according to sites capacity, treatment strategy and gender. **Table S2a.** Percentage % of patients received Oral antidiabetic agents OADs. b Percentage % of patients received any Injectable treatments, not including Insulin. c Percentage % of patients received Insulin treatment. d Daily dose of treatment (total). e Daily dose per treatment strategy. **Table S3.** Risk Factors per treatment strategy at current period. **Table S4.** Distribution of FPG values across < 70 mg/dl, 70-100 mg/dl, 100-130 mg/dl and ≥ 130 mg/dl at initial diagnosis and current period per treatment strategy. **Table S5.** Difference of laboratory measurements (HbA1c & FPG) between initial diagnosis and current period. **Table S6.** Assessment of patients’ compliance, interest and active participation per treatment strategy at current period. **Table S7.** QoL score -scale from 1 to 10 (Worst to Best) at initial diagnosis and current period per treatment strategy (*p* <  0.001). **Table S8.** Doctors’ specialty, geographical region and hospital management per treatment strategy at current period. (DOCX 68 kb)

## Abbreviations

AACE: American Association of Clinical Endocrinologists
ADA: American Diabetes Association
AEs: Adverse Events
EASD: European Diabetes Association
GCPs: Good Pharmacoepidemiology Practices
HAD: Hellenic Diabetes Association
HDL-C: High Density Lipoprotein-Cholesterol
HES: Hellenic Endocrine Society
IDF: International Diabetes Federation
IQR: Interquartile Range
LDL-C: Low Density Lipoprotein-Cholesterol
NGDA: Northern Greece Diabetes Association
NHS: National Health System
OADs: Oral antidiabetic drugs
QoL: Quality of Life
SD: Standard Deviation
T2DM: Type-2 Diabetes Mellitus
