# Supplementary material for: A Greek registry of current type 2 diabetes management, aiming to determine core clinical approaches, patterns and strategies
Source: BMC Endocr Disord. 2019 Apr 25;19:39. doi: 10.1186/s12902-019-0364-5 (PMC6482543; doi:10.1186/s12902-019-0364-5)
Supplement: Supplementary file 1 — AGREEMENT Investigators. Table S1. Stratification of patients according to sites capacity, treatment strategy and gender. Table S2a. Percentage % of patients received Oral antidiabetic agents OADs. b Percentage % of patients received any Injectable treatments, not including Insulin. c Percentage % of patients received Insulin treatment. d Daily dose of treatment (total). e Daily dose per treatment strategy. Table S3. Risk Factors per treatment strategy at current period. Table S4. Distribution of FPG values across < 70 mg/dl, 70-100 mg/dl, 100-130 mg/dl and ≥ 130 mg/dl at initial diagnosis and current period per treatment strategy. Table S5. Difference of laboratory measurements (HbA1c & FPG) between initial diagnosis and current period. Table S6. Assessment of patients’ compliance, interest and active participation per treatment strategy at current period. Table S7. QoL score -scale from 1 to 10 (Worst to Best) at initial diagnosis and current period per treatment strategy (p < 0.001). Table S8. Doctors’ specialty, geographical region and hospital management per treatment strategy at current period. (DOCX 68 kb) [file 12902_2019_364_MOESM1_ESM.docx]

**Supplementary Appendix**

This appendix has been provided by the authors to give readers additional information about their work.

***AGREEMENT Investigators**

1. Alexiou Zoi, Second Department of Internal Medicine, General Hospital of Elefsina Thriasio, Elefsina, Greece.
2. Almanidou-Kougioumtzidi Olga, Second Department of Internal Medicine, General Hospital of Karditsa, Karditsa, Greece.
3. Anastasiou Eleni, Department of Endocrinology & Diabetes Center, General Hospital of Athens "Alexandra", Athens, Greece.
4. Avramidis Iakovos, First Department of Internal Medicine, General Hospital of Thessaloniki "G.Papanikolaou", Thessaloniki, Greece.
5. Βakidis Sofoklis, Department of Internal Medicine, General Hospital of Lakonia – Molaoi, Molaoi, Greece.
6. Bargiota Alexandra, Department of Endocrinology and Metabolic Diseases, University Hospital of Larisa, Larisa, Greece.
7. Bikas Christos, Department of Internal Medicine, General Hospital of Pyrgos, Pyrgos, Greece.
8. Bousboulas Stavros, Diabetes Centre, General Hospital of Nikea-Piraeus "Agios Panteleimon", Piraeus, Greece.
9. Boutel Dimitrios, Department of Internal Medicine, General Hospital of Giannitsa, Giannitsa, Greece.
10. Chaliotis Georgios, Department of Internal Medicine, General Hospital of Chalkida, Chalkida, Greece.

Chrisoulidou Alexandra, Department of Endocrinology, Theagenio Cancer Hospital, Thessaloniki, Greece.

Didangelos Triantafyllos, Diabetes Center, First Propaedeutic Department of Internal Medicine, Medical School, Aristotle University of Thessaloniki, AHEPA Hospital, Thessaloniki, Greece.

Dimitriadis George, Second Department of Internal Medicine and Research Institute and Diabetes Center, Attikon University Hospital, Athens, Greece.

Dimou Eftihia, Department of Internal Medicine, General Hospital of Samos "Agios Panteleimon", Samos ,Greece.

Douitsis Petros, Department of Internal Medicine, “Eleni Dimitriou” General Hospital of Florina, Florina, Greece.

Doupis John, Internal Medicine and Diabetes Department, Salamis Naval Hospital, Salamis, Greece.

Exiara Triada, Department of Internal Medicine, General Hospital of Komotini, Komotini, Greece.

Gkioka Maria, Department of Internal Medicine, General Hospital of Patras "Agios Andreas", Patras, Greece.

Gkioulos Nikolaos, Health Center, Ag. Nikolaos, General Hospital of Kalamata, Kalamata, Greece.

Grigoropoulou Pinelopi, Department of Internal Medicine, General Hospital of Athens "Elpis", Athens, Greece.

Ioannidis Ioannis, Diabetes and Obesity Outpatient Clinics, General Hospital of Nea Ionia “Konstantopouleio-Patission”, Athens, Greece.

Kirlaki Evridiki, Diabetes Center, General Hospital of Heraklio "Venizeleio-Pananio", Heraklio, Greece.

Kitsios Kostas, Diabetes Outpatient Clinics, General Hospital of Thessaloniki "G.Gennimatas - Ag.Dimitrios", Thessaloniki, Greece.

Kokkoris Panagiotis, Department of Endocrinology and Diabetes, 251 Hellenic Air Force & VA General Hospital, Athens, Greece.

Kotsa Kalliopi, First Department of Internal Medicine, Division of Endocrinology and Metabolism, AHEPA Hospital, Aristotle University of Thessaloniki, Thessaloniki, Greece.

Kouroglou Maria, Department of Internal Medicine, General Hospital of Mytilene "Vostanio", Mytilene, Greece.

Lanaras Leonidas, Department of Internal Medicine, General Hospital of Lamia, Lamia, Greece.

Magiannis Konstantinos, Department of Internal Medicine, General Hospital of Grevena, Grevena, Greece.

Manes Christos, Diabetes Center, Papageorgiou General Hospital, Thessaloniki, Greece.

Marathonitis Georgios, First Department of Internal Medicine, General Hospital of Attiki "Sismanoglio - Amalia Fleming" - Hospital Unit "Amalia Fleming", Melissia, Greece.

Melidonis Andreas, Diabetes Center, Tzanio General Hospital, Piraeus, Greece.

Migdalis Ilias, Second Medical Department and Diabetes Centre, NIMTS Hospital, Athens, Greece.

Mitrakou Asimina, Department of Clinical Therapeutics, Athens University Medical School, General Hospital of Athens "Alexandra", Athens, Greece.

Papantoniou Stefanos, Diabetes Clinic, First Department of Internal Medicine, General Hospital of Kavala, Kavala, Athens, Greece.

Papazoglou Dimitrios, Diabetes Centre, Second Department of Internal Medicine, University General Hospital of Alexandroupolis, Democritus University of Thrace, Alexandroupolis, Greece.

Piaditis Georgios, Department of Endocrinology and Diabetes Center, “G. Gennimatas”, General Hospital of Athens, Athens, Greece.

Potolidis Evangelos, Department of Internal Medicine, General Hospital of Volos "Achillopoulio", Volos, Greece.

Prokovas Ioannis, Endocrinology Clinic, General Hospital of Nikea-Piraeus "Agios Panteleimon", Piraeus, Greece.

Rizos Evangelos, Second Department of Internal Medicine, University Hospital of Ioannina, Ioannina, Greece.

Rogkoti Maria, Department of Internal Medicine, General Hospital of Thessaloniki "G.Gennimatas - Ag.Dimitrios" - Unified Hospital "Agios Dimitrios", Thessaloniki, Greece.

Romanidou Alexandra, First Department of Internal Medicine, General Hospital of Serres, Serres, Greece.

Sampanis Christos, Diabetes Centre, Second Propaedeutic Department of Internal Medicine, General Hospital of Thessaloniki - "Hippokration", Thessaloniki, Greece.

Satsoglou Aimilios, Department of Internal Medicine, General Hospital of Goumenissa, Kilkis, Greece.

Simelidis Dimitrios, Department of Internal Medicine, Mamatseio General Hospital of Kozani, Kozani, Greece.

Taisir El Hasban, Health Center of Elefsina, General Hospital of Elefsina Thriasio, Elefsina, Greece.

Tentolouris Nikolaos, First Department of Propaedeutic Medicine, Diabetes Center, Athens University Medical School, Laiko Hospital, Athens, Greece.

Thanopoulou Anastasia, Second Department of Internal Medicine, General Hospital “Hippocratio”, National and Kapodistrian University, Athens, Greece.

Tolis Apostolos, Second Department of Internal Medicine, “G. Gennimatas”, General Hospital of Athens, Athens, Greece.

Tsanikidis Iraklis, Department of Internal Medicine, General Hospital of Katerini, Katerini, Greece.

Tsapas Apostolos, Clinical Research and Evidence Based Medicine Unit, Second Medical Department, Aristotle University of Thessaloniki, General Hospital of Thessaloniki “Hippokration”, Thessaloniki, Greece.

Tsapogas Panagiotis, Department of Internal Medicine, General Hospital of Corfu, Corfu, Greece.

Tsatsoulis Agathoklis, Department of Endocrinology, University Hospital of Ioannina, Ioannina, Greece.

Tsotoulidis Stefanos, Department of Internal Medicine, General Hospital of Chalkidiki – Kassandria, Kassandria, Greece.

Tzatzagou Glykeria, First Department of Internal Medicine, Papageorgiou General Hospital, Thessaloniki, Greece.

Vasiliadis Panagiotis, Department of Internal Medicine, General Hospital of Thessaloniki "G. Papanikolaou" - Interconnected Psychiatric Hospital of Thessaloniki, Thessaloniki, Greece.

Vasilopoulos Charalampos, Department of Endocrinology, Diabetes and Metabolism, “Evangelismos” Hospital, Athens, Greece.

Vlachogiannis Anestis, Department of Internal Medicine, General Hospital of Drama, Drama, Greece.

Vryonidou Andromachi, Department of Endocrinology and Diabetes, Hellenic Red Cross Hospital, Athens, Greece.

Xilomenos Apostolos, Department of Internal Medicine, General Hospital of Agrinio, Agrinio, Greece.

**Table S1 Stratification of patients according to sites capacity, treatment strategy and gender**

| **Sites capacity** | **No of sites** | **Treatment strategy A** | **Treatment strategy B** | **Treatment strategy C** | **Sum of patients at each site**   \|  \| \| --- \| | **Sum of patients in the study** |
| --- | --- | --- | --- | --- | --- | --- | --- |
| **A** | 18 | 6 (3M+3W) | 14 (7M+7W) | 14 (7M+7W) | 34 (17M/17W) | 612 |
| **B** | 19 | 4 (2M+2W) | 6 (3M+3W) | 6 (3M+3W) | 16 (8M/8W) | 304 |
| **C** | 32 | 2 (1M+1W) | 4 (2M+2W) | 4 (2M+2W) | 10 (5M/5W) | 320 |
| **In total** | 69 | 12  (6M+6W) | 24 (12M+12W) | 24 (12M+12W) | 60 (30M/30W) | 1236 |

M-Men, W-Women

**Table S2a. Percentage % of patients receiving Oral antidiabetic agents OADs**

| **OADs** | **N (N%)** | **Treatment Category** | | | **Total** |
| --- | --- | --- | --- | --- | --- |
|  |  | **A (N=241)** | **B (N=478)** | **C (N=470)** |  |
| Biguanides: Metformin | **N** | 200 | 439 | 320 | 959 |
|  | **N%** | 82.9 | 91.8 | 68.0 |  |
| Alpha-glucosidase inhibitors: Acarbose | **N** | 0 | 5 | 1 | 6 |
|  | **N%** | 0 | 1.0 | 0.2 |  |
| Sulphonylureas: Glibenclamide | **N** | 0 | 2 | 2 | 4 |
|  | **N%** | 0 | 0.4 | 0.4 |  |
| Sulphonylureas: Gliclazide MR | **N** | 7 | 74 | 28 | 109 |
|  | **N%** | 2.9 | 15.5 | 5.9 |  |
| Sulphonylureas: Glimepiride | **N** | 2 | 129 | 58 | 189 |
|  | **N%** | 0.8 | 27.0 | 12.3 |  |
| Meglitinides: Repaglinide | **N** | 0 | 8 | 11 | 19 |
|  | **N%** | 0 | 1.7 | 2.3 |  |
| Meglitinides: Nateglinide | **N** | 1 | 11 | 2 | 14 |
|  | **N%** | 0.4 | 2.3 | 0.4 |  |
| Glitazones: Pioglitazone | **N** | 2 | 54 | 13 | 69 |
|  | **N%** | 0.8 | 11.3 | 2.7 |  |
| DPP-4 inhibitors: Sitagliptin | **N** | 8 | 128 | 54 | 190 |
|  | **N%** | 3.3 | 26.8 | 11.5 |  |
| DPP-4 inhibitors: Vildagliptin | **N** | 5 | 178 | 70 | 253 |
|  | **N%** | 2.1 | 37.2 | 14.9 |  |
| DPP-4 inhibitors: Saxagliptin | **N** | 2 | 18 | 3 | 23 |
|  | **N%** | 0.8 | 3.8 | 0.6 |  |
| Other Oral antidiabetic agent 1 | **N** | 0 | 23 | 14 | 37 |
|  | **N%** | 0 | 4.8 | 3.0 |  |
| Other Oral antidiabetic agent 2 | **N** | 2 | 4 | 1 | 7 |
|  | **N%** | 0.8 | 0.8 | 0.2 |  |

**Table S2b. Percentage % of patients receiving any Injectable treatments, not including Insulin**

| **Non Insulin** | **N (N%)** | **Treatment Category** | | **Total** |
| --- | --- | --- | --- | --- |
|  |  | **B (N=478)** | **C (N=470)** |  |
| GLP-1 Mimetics (GLP-1 Receptor Agonists): Exenatide | **N** | 0 | 5 | 5 |
|  | **N%** | 0 | 1.1 |  |
| GLP-1 Mimetics (GLP-1 Receptor Agonists): Liraglutide | **N** | 49 | 48 | 97 |
|  | **N%** | 10.2 | 10.2 |  |
| GLP-1 Mimetics (GLP-1 Receptor Agonists): Lixisenatide | **N** | 4 | 12 | 16 |
|  | **N%** | 0.8 | 2.6 |  |
| Other injectable agent (excluding Insulin regimens) | **N** | 2 | 0 | 2 |
|  | **N%** | 0.4 | 0 |  |

**Table S2c. Percentage % of patients receiving Insulin treatment**

| **Insulin** | **N (N%)** | **Treatment Category** |
| --- | --- | --- |
|  |  | **C (N=470)** |
| Insulin Regular | **N** | 5 |
|  | **N%** | 1.1 |
| Insulin Actrapid | **N** | 16 |
|  | **N%** | 3.5 |
| Insulin Lispro | **N** | 31 |
|  | **N%** | 6.6 |
| Insulin Aspart | **N** | 54 |
|  | **N%** | 11.5 |
| Insulin Glulisine | **N** | 48 |
|  | **N%** | 10.2 |
| Insulin NPH | **N** | 1 |
|  | **N%** | 0.2 |
| Insulin Protaphan | **N** | 2 |
|  | **N%** | 0.4 |
| Basal Insulin Glargine | **N** | 319 |
|  | **N%** | 67.8 |
| Basal Insulin Detemir | **N** | 39 |
|  | **N%** | 8.3 |
| 30%Fast / 70% Intermediate | **N** | 29 |
|  | **N%** | 6.2 |
| 40% Fast / 60% Intermediate | **N** | 0 |
|  | **N%** | 0 |
| 50% Fast / 50% Intermediate | **N** | 1 |
|  | **N%** | 0.2 |
| Biphasic Insulin Aspart 30/70 | **N** | 35 |
|  | **N%** | 7.4 |
| Biphasic Insulin Lispro 25/75 | **N** | 25 |
|  | **N%** | 5.3 |
| Biphasic Insulin Lispro 50/50 | **N** | 1 |
|  | **N%** | 0.2 |
| Other basal Insulin | **N** | 17 |
|  | **N%** | 3.6 |

**Table S2d. Daily dose of treatment (total)**

| **Treatment** | **N** | **Mean Dose** |
| --- | --- | --- |
|  |  |  |
| Daily dose Metformin (mg) | 959 | 1753 |
| Daily dose Acarbose (mg) | 6 | 167 |
| Daily dose Glibenclamide (mg) | 4 | 6 |
| Daily dose Gliclazide MR (mg) | 109 | 51 |
| Daily dose Glimepiride (mg) | 189 | 3.4 |
| Daily dose Repaglinide (mg) | 19 | 3.4 |
| Daily dose Nateglinide (mg) | 14 | 240 |
| Daily dose Pioglitazone (mg) | 69 | 27 |
| Daily dose Sitagliptin (mg) | 190 | 105 |
| Daily dose Vildagliptin (mg) | 253 | 98 |
| Daily dose Saxagliptin (mg) | 23 | 5 |
| Daily dose Exenatide (mg) | 5 | 20 |
| Daily dose Liraglutide (mg) | 97 | 1 |
| Daily dose Lixisenatide (mg) | 16 | 19 |
| Daily dose Insulin Regular (IU) | 5 | 34 |
| Daily dose Insulin Actrapid (IU) | 16 | 111 |
| Daily dose Insulin Lispro (IU) | 31 | 52 |
| Daily dose Insulin Aspart (IU) | 54 | 34 |
| Daily dose Insulin Glulisine (IU) | 48 | 64 |
| Daily dose Insulin NPH (IU) | 1 | 8 |
| Daily dose Insulin Protaphan (IU) | 2 | 29 |
| Daily dose Basal Insulin Glargine (IU) | 319 | 30 |
| Daily dose Basal Insulin Detemir (IU) | 39 | 36 |
| Daily dose 30% Fast / 70% Intermediate (IU) | 29 | 95 |
| Daily dose 40% Fast / 60% Intermediate (IU) | 0 | . |
| Daily dose 50% Fast / 50% Intermediate (IU) | 1 | 50 |
| Daily dose Biphasic Insulin Aspart 30/70 (IU) | 35 | 83 |
| Daily dose Biphasic Insulin Lispro 25/75 (IU) | 25 | 97 |
| Daily dose Biphasic Insulin Lispro 50/50 (IU) | 1 | 124 |
| Daily dose Other basal Insulin (IU) | 17 | 38 |

**Table S2e. Daily dose per treatment strategy**

| **Treatment** | **Treatment Category** | | | | | | | | |
| --- | --- | --- | --- | --- | --- | --- | --- | --- | --- |
|  | **A** | | | **B** | | | **C** | | |
|  | N | Mean Dose |  | N | Mean Dose |  | N | Mean Dose |  |
| Daily dose Metformin (mg) | 200 | 1535 |  | 439 | 1819 |  | 320 | 1800 |  |
| Daily dose Acarbose (mg) |  |  |  | 5 | 160 |  | 1 | 200 |  |
| Daily dose Glibenclamide (mg) |  |  |  | 2 | 8 |  | 2 | 5 |  |
| Daily dose Gliclazide MR (mg) | 7 | 56 |  | 74 | 52 |  | 28 | 50 |  |
| Daily dose Glimepiride (mg) | 2 | 2 |  | 129 | 3 |  | 58 | 3 |  |
| Daily dose Repaglinide (mg) |  |  |  | 8 | 3 |  | 11 | 4 |  |
| Daily dose Nateglinide (mg) | 1 | 360 |  | 11 | 207 |  | 2 | 360 |  |
| Daily dose Pioglitazone (mg) | 2 | 30 |  | 54 | 27 |  | 13 | 29 |  |
| Daily dose Sitagliptin (mg) | 8 | 113 |  | 128 | 105 |  | 54 | 104 |  |
| Daily dose Vildagliptin (mg) | 5 | 70 |  | 178 | 100 |  | 70 | 94 |  |
| Daily dose Saxagliptin (mg) | 2 | 5 |  | 18 | 5 |  | 3 | 5 |  |
| Daily dose Exenatide (mg) |  |  |  |  |  |  | 5 | 20 |  |
| Daily dose Liraglutide (mg) |  |  |  | 49 | 1 |  | 48 | 2 |  |
| Daily dose Lixisenatide (mg) |  |  |  | 4 | 18 |  | 12 | 19 |  |
| Daily dose Insulin Regular (IU) |  |  |  |  |  |  | 5 | 34 |  |
| Daily dose Insulin Actrapid (IU) |  |  |  |  |  |  | 16 | 111 |  |
| Daily dose Insulin Lispro (IU) |  |  |  |  |  |  | 31 | 52 |  |
| Daily dose Insulin Aspart (IU) |  |  |  |  |  |  | 54 | 34 |  |
| Daily dose Insulin Glulisine (IU) |  |  |  |  |  |  | 48 | 64 |  |
| Daily dose Insulin NPH (IU) |  |  |  |  |  |  | 1 | 8 |  |
| Daily dose Insulin Protaphan (IU) |  |  |  |  |  |  | 2 | 29 |  |
| Daily dose Basal Insulin Glargine (IU) |  |  |  |  |  |  | 319 | 30 |  |
| Daily dose Basal Insulin Detemir (IU) |  |  |  |  |  |  | 39 | 36 |  |
| Daily dose 30% Fast / 70% Intermediate (IU) |  |  |  |  |  |  | 29 | 95 |  |
| Daily dose 40% Fast / 60% Intermediate (IU) |  |  |  |  |  |  | 0 |  |  |
| Daily dose 50% Fast / 50% Intermediate (IU) |  |  |  |  |  |  | 1 | 50 |  |
| Daily dose Biphasic Insulin Aspart 30/70 (IU) |  |  |  |  |  |  | 35 | 83 |  |
| Daily dose Biphasic Insulin Lispro 25/75 (IU) |  |  |  |  |  |  | 25 | 97 |  |
| Daily dose Biphasic Insulin Lispro 50/50 (IU) |  |  |  |  |  |  | 1 | 124 |  |
| Daily dose Other basal Insulin (IU) |  |  |  |  |  |  | 17 | 38 |  |

For Metformin the medians and the distributions across Treatment Categories differ statistical significant (Median Test and Kruskal-Wallis Test p-value <0,05).

The distribution of the Daily dose of Vildagliptin across Treatment Categories differs statistical significant (Kruskal-Wallis Test p-value =0,001).

The distribution of the Daily dose of Liraglutide is not the same across Treatment Categories and the difference is statistical significant (Kruskal-Wallis Test p-value =0,039).

**Table S3 Risk Factors per treatment strategy at current period**

| **Risk Factor n (%)** | **Total** |  | **Treatment Strategy** | | | **P** |
| --- | --- | --- | --- | --- | --- | --- |
|  |  |  | **A** | **B** | **C** |  |
| **Western lifestyle** | 197 (16.6%) |  | 40 (16.6%) | 78 (16.3%) | 79 (16.8%) | 0.979 |
| **Sedentary work** | 167 (14%) |  | 34 (14.1%) | 65 (13.6%) | 68 (14.5%) | 0.928 |
| **Limited physical exercise** | 576 (47.5%) |  | 102 (42.3%) | 223 (46.7%) | 251 (53.4%) | **0.012** |
| **Increased body weight** | 390 (32.9%) |  | 81 (33.6%) | 159 (33.3%) | 150 (31.9%) | 0.867 |
| **Obesity** | 158 (39.1%) |  | 84 (34.9%) | 188 (39.3%) | 202 (43.0%) | 0.106 |
| **Family history of T1DM** | 11 (0.9%) |  | 1 (0.4%) | 6 (1.3%) | 4 (0.9%) | 0.527 |
| **Family history of T2DM** | 577 (47.4%) |  | 101 (41.9%) | 240 (50.2%) | 236 (50.2%) | 0.071 |
| **Overall poor diet** | 219 (18%) |  | 38 (15.8%) | 71 (14.9%) | 110 (23.4%) | **0.002** |
| **Diet rich in carbohydrates with high glycaemic index** | 128 (10.8%) |  | 21 (8.7%) | 47 (9.8%) | 60 (12.8%) | 0.178 |
| **Diet rich in sugars** | 113 (9.5%) |  | 18 (7.5%) | 47 (9.8%) | 48 (10.2%) | 0.474 |
| **High-fat diet, particularly saturated** | 136 (11.4%) |  | 27 (11.2%) | 53 (11.1%) | 56 (11.9%) | 0.916 |
| **Smoking** | 168 (14.1%) |  | 35 (14.5%) | 63 (13.2%) | 70 (14.9%) | 0.736 |
| **Alcohol consumption** | 55 (4.6%) |  | 10 (4.1%) | 30 (6.3%) | 15 (3.2%) | 0.072 |
| **Other** | 13 (1.1%) |  | 4 (1.7%) | 5 (1.0%) | 4 (0.9%) | 0.613 |

**Table S4** **Distribution of FPG values across <70 mg/dl, 70-100mg/dl, 100-130mg/dl and ≥130mg/dl at** **initial diagnosis and current period per treatment strategy**

| **FPG mg/dl** | **Initial diagnosis** | | | | | **Current period** | | | | |
| --- | --- | --- | --- | --- | --- | --- | --- | --- | --- | --- |
|  | **Treatment Strategy** | | | | | **Treatment Strategy** | | | | **P** |
|  | **All** | **A** | **B** | **C** | | **All** | **A** | **B** | **C** |  |
|  |  |  |  | |  |  |  |  |  |  |
| **Full analysis set, n** | 827 (100%) | 520 (62.8%) | 218 (26.4%) | | 89 (10.8%) | 1108 (100%) | 223 (20.1%) | 445 (40.2%) | 440 (39.7%) |  |
| **<70** | 0 (0%) | 0 (0%) | 0 (0%) | | 0 (0%) | 6 (0.5%) | 0 (0%) | 1 (0.2%) | 5 (1.0%) | < 0.001^(1)^ |
| **[70-100)** | 7 (0.8%) | 6 (1.0%) | 0 (0%) | | 1 (1.0%) | 135 (12.0%) | 28 (12.0%) | 54 (12.0%) | 53 (12.0%) |  |
| **[100-130)** | 76 (09.0%) | 60 (11.0%) | 11 (5.0%) | | 5 (5.0%) | 469 (42.0%) | 121 (54.00%) | 193 (43.0%) | 155 (35.0%) |  |
| **[70-130]** | 83 (10.0%) | 66 (12.0%) | 11 (5.0%) | | 6 (6.0%) | 604 (54.0%) | 149 (66.0%) | 248 (55.0%) | 213 (48.0%) |  |
| **>=130** | 744 (90.0%) | 454 (87.0%) | 207 (94.0%) | | 83 (93.0%) | 498 (44.0%) | 74 (33.0%) | 197 (44.0%) | 227 (51.0%) |  |
|  |  |  |  | |  |  |  |  |  |  |

1. Chi – square independence test,

**Table S5** **Difference of laboratory measurements (HbA1c & FPG) between initial diagnosis and current period**

| **Laboratory measurements** | | **N** | **Diff<0** | **Diff>0** | **Median Difference** | **95% CI of difference** | **Wilcoxon text, p** |
| --- | --- | --- | --- | --- | --- | --- | --- |
| **HbA1c %** | | | | | | | |
| **Treatment strategy** | **A** | 153 | 29 | 109 | -0.70 | (-0.95 , -0.55 ) | <0.001 |
|  | **B** | 230 | 32 | 195 | -1.25 | (-1.50 , -1.05) | <0.001 |
|  | **C** | 168 | 26 | 136 | -1.50 | (-1.85 , -1.20) | <0.001 |
|  | **Total** | 551 | 87 | 440 | -1.15 | (-1.30 , -1.05) | <0.001 |
| **FBG mg/dl** | | | | | | | |
| **Treatment strategy** | **A** | 185 | 39 | 142 | -32.00 | (-40.5, - 25.0) | <0.001 |
|  | **B** | 327 | 46 | 276 | -54.50 | (-61.5, -47.5) | <0.001 |
|  | **C** | 288 | 31 | 251 | -80.00 | (-91.0, -69.5) | <0.001 |
|  | **Total** | 800 | 107 | 669 | -57.00 | (-62.5, -52.0) | <0.001 |

**Table S6** **Assessment of patients’ compliance, interest and active participation per treatment strategy at current period**

|  | **Treatment strategy** | | | | **P** |
| --- | --- | --- | --- | --- | --- |
|  | **A** | **B** | **C** | **Total** |  |
| **Compliance to Physicians‘Treatment Instructions n (%)** | | | | | |
| **Variable** | 40 (16.6%) | 116 (24.4%) | 163 (34.7%) | 319 (26.9%) | <0.001 |
| **Generally judged** | 186 (77.2%) | 335 (70.4%) | 264 (56.2%) | 785 (66.1%) |  |
| **Generally poor** | 15 (6.2%) | 25 (5.3%) | 43 (9.1%) | 83 (7.0%) |  |
| **Patients Interest and Active Participation in the treatment of the disease n (%)** | | | | | |
| **Variable** | 29 (12.0%) | 99 (20.8%) | 133 (28.3%) | 261 (22.0%) | <0.001 |
| **Satisfactory** | 198 (82.2%) | 357 (75.0%) | 298 (63.4%) | 853 (71.9%) |  |
| **Indifferent** | 7 (2.9%) | 7 (1.5%) | 15 (3.2%) | 29 (2.4%) |  |
| **Inadequate** | 7 (2.9%) | 13 (2.7%) | 24 (5.1%) | 44 (3.7%) |  |

**Table S7** **QoL score -scale from 1 to 10 (Worst to Best) at initial diagnosis and current period per treatment strategy (p<0.001)**

|  | | **Initial diagnosis** | | |
| --- | --- | --- | --- | --- |
|  |  | **Treatment strategy** | | |
|  |  | **A** | **B** | **C** |
| **QoL** | **Median (IQR)** | 7 (5-9) | 7(5-9) | 7 (5-9) |
|  | | **Current period** | | |
|  |  | **Treatment strategy** | | |
|  |  | **A** | **B** | **C** |
| **QoL** | **Median (IQR)** | 8(7-9) | 8(7-9) | 7(5-9) |

**Table S8** **Doctors’ specialty, geographical region and hospital management per treatment strategy at current period**

| **Sites characteristics** | **Treatment Strategy** | | | **P** |
| --- | --- | --- | --- | --- |
|  | **A** | **B** | **C** |  |
| **Doctor Specialty n (%)** |  |  |  |  |
| **Endocrinologist** | 20 (8.3%) | 42 (8.8%) | 42 (8.9%) | 0.99 |
| **Diabetologist** | 217 (90.0%) | 430 (90.0%) | 422 (89.8%) |  |
| **Internist** | 4 (1.7%) | 6 (1.3%) | 6 (1.3%) |  |
| **Geographical region n (%)** |  |  |  |  |
| **Attiki** | 71 (29.5%) | 154 (32.2%) | 148 (31.5%) | 0.997 |
| **Piraeus and Aegean Island** | 26 (10.8%) | 59 (12.3%) | 55 (11.7%) |  |
| **Makedonia and Thraki** | 86 (35.7%) | 161 (33.7%) | 163 (34.7%) |  |
| **Thessaly** | 18 (7.5%) | 34 (7.1%) | 34 (7.2%) |  |
| **Peloponissos** | 34 (14.1%) | 56 (11.7%) | 56 (11.9%) |  |
| **Crete** | 6 (2.5%) | 14 (2.9%) | 14 (3.0%) |  |
| **Hospital management n (%)** | | | | |
| **General Hospital** | 191 (79.3%) | 375 (78.5%) | 371 (78.9%) | 0.989 |
| **University Hospital** | 36 (14.9%) | 74 (15.5%) | 74 (15.7%) |  |
| **Military Hospital** | 14 (5.8%) | 29 (6.1%) | 25 (5.3%) |  |
